# Understanding Robustness and Generalization of Artificial Neural Networks Through Fourier Masks

**DOI:** 10.3389/frai.2022.890016

**Published:** 2022-07-12

**Authors:** Nikos Karantzas, Emma Besier, Josue Ortega Caro, Xaq Pitkow, Andreas S. Tolias, Ankit B. Patel, Fabio Anselmi

**Affiliations:** ^1^Department of Neuroscience, Baylor College of Medicine, Houston, TX, United States; ^2^Center for Neuroscience and Artificial Intelligence, Baylor College of Medicine, Houston, TX, United States; ^3^Department of Electrical and Computer Engineering, Rice University, Houston, TX, United States

**Keywords:** Fourier analysis, symmetry, robustness, generalization, neural networks, data augmentation

## Abstract

Despite the enormous success of artificial neural networks (ANNs) in many disciplines, the characterization of their computations and the origin of key properties such as generalization and robustness remain open questions. Recent literature suggests that robust networks with good generalization properties tend to be biased toward processing low frequencies in images. To explore the frequency bias hypothesis further, we develop an algorithm that allows us to learn *modulatory masks* highlighting the *essential input frequencies* needed for preserving a trained network's performance. We achieve this by imposing *invariance* in the loss with respect to such modulations in the input frequencies. We first use our method to test the low-frequency preference hypothesis of adversarially trained or data-augmented networks. Our results suggest that adversarially robust networks indeed exhibit a low-frequency bias but we find this bias is also dependent on directions in frequency space. However, this is not necessarily true for other types of data augmentation. Our results also indicate that the essential frequencies in question are effectively the ones used to achieve generalization in the first place. Surprisingly, images seen through these modulatory masks are not recognizable and resemble texture-like patterns.

## 1. Introduction

Artificial neural networks (ANNs) have achieved impressive performance in a variety of tasks, e.g., object recognition, function approximation, natural language processing, etc. (LeCun et al., 2015). However, their computational capacity remains rather opaque. In particular, the operations performed by ANNs are profoundly constrained by the choice of architecture, initialization, optimization techniques, etc., and such constraints have a significant impact on key properties such as generalization power and robustness. Studying adversarial robustness has been a very active area of research, since it is closely related to how trustworthy and reliable neural networks can be (Goodfellow et al., 2014). One of the most explored directions has been the analysis of adversarial perturbations from a frequency standpoint. For example, the work of Yin et al. (2019) establishes a relationship between the frequency domain of different noises (e.g Adversarial examples and Common corruptions) and model performance. In particular, they show that deep neural networks are more sensitive to high frequency adversarial attacks or common corruptions such as random noise, contrast change, and blurring. Additionally, adversarial perturbations of commonly trained models tend to be higher frequency than their adversarially trained counterparts. Furthermore, (Wang et al., 2020) found that high frequency features are necessary for good generalization performance while the work of Sharma et al. (2019) shows that performance improvements in white-box and black-box transfer settings can be achieved only when low frequency components are preserved.

These results have led to various methodologies that help us understand artificial neural networks through a frequency lens. One such method is Neural Anisotropic Directions (NADs) (Ortiz-Jimenez et al., 2020a,b). NADs are input directions for which a network is able to linearly classify data. Furthermore, Tsuzuku and Sato (2019) introduced a method to compute a neural network's sensitivity to input directions in the Fourier domain. Moreover, Li et al. (2022) show that robust deep learning object recognition models rely on low frequency information in natural images. Finally, Abello et al. (2021) divides the image frequency spectrum into disjoint disks and provides evidence that mid or high-level frequencies are important for ANN classification.

In this work we introduce a simple and easy-to-use method to *learn* the input frequency features that a network deems essential in order to achieve its classification performance. We visualize the relevant frequencies by learning a *modulatory mask* on the Fourier transform of the input data that defines a modulation-invariant loss function obtained *via* a simple optimization algorithm (Section 2.1). We compare such masks with their adversarially trained or data augmented counterparts (Section 3). In the case of adversarial training, the comparison is done at two levels of analysis. At a global level, we learn a mask for the entire test set. Our goal is to find the frequencies that allow for *robust generalization*. At a single image level, we explore the frequencies responsible for adversarial success/failure. Those comparisons allow us to test the hypothesis that adversarially trained models have a bias toward low frequency features and assess if the same holds for other types of data augmentation.

In the case of adversarial augmentation, our results confirm the low frequency bias hypothesis. However, they also highlight that the important frequency redistribution due to the augmentation is highly anisotropic. In the case of common data augmentations instead, our results show how the frequency reorganization depends on the type of augmentation, e.g., rotation- or scale-augmented models exhibit mid-high and low frequency biases, respectively.

The single-image mask analysis reveals that only a few, class-specific frequencies are crucial to determine a network's decision. Moreover, *those frequencies are effectively the ones used to achieve its performance*. In fact, mask-filtered images do not alter performance at all. However, surprisingly, they are not recognizable. They are characterized by texture-like patterns. This is in line with previous work by Geirhos et al. (2019), which provided evidence that Convolutional Neural Networks (CNNs) are biased toward textures rather than shapes in object recognition. Our method differs from all previous ones in that we explicitly learn the frequencies defining the features a model is sensitive to.

## 2. Methods

### 2.1. Approach

Artificial neural networks and their associated task-dependent losses define highly non-linear functions of their input. In terms of the frequency content found in a signal, the effect of the application of a non-linear function can be understood by considering the following simple one-dimensional example. Suppose *f*(*t*) = cos(*w*_1_*t*)+cos(*w*_2_*t*) is a sound wave and let σ(*t*) = *t*^2^. Then


$$\begin{array}{l} (\sigma \circ f)(t)=\frac{1}{2}[2+\cos(2w_{1}t)+\cos(2w_{2}t)+2\cos((w_{1}+w_{2})t)\;\\ \;+2\cos((w_{1}-w_{2})t)]. \end{array}$$

**Figure 1:**
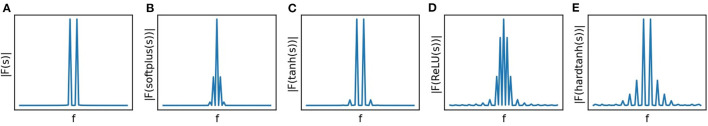
Non-linear distortions in the frequency domain due to the application of **(B)** softplus, **(C)** tanh, **(D)** ReLU, and **(E)** hardtanh non-linear activations on *s*(·) = sin(·) **(A)**.

We see that one of the effects of σ on *f* is to generate the new frequency components *w*_1_−*w*_2_, *w*_1_+*w*_2_, 2*w*_1_, 2*w*_2_. The first two are due to a phenomenon called *intermodulation*, the last are due to what is called *harmonic distortion*. Harmonic distortion has been studied in the context of neural networks with different activation functions by Christian et al. (2021), where an empirical demonstration and theoretical arguments are given to support the claim that the presence of non-linear elements mainly causes a spread in the frequency content of the loss function. Their reasoning is the following: let *ϕ*: ℝ → ℝ be a non-linear function and *Tϕ* denote its Taylor expansion around the origin. For *x* ∈ ℝ^*d*^, using the convolution theorem yields


(1) $$\begin{array}{r} \begin{array}{c} FT\phi (x)=F\sum_{n}a_{n}\underset{\text{n-times}}{\underset{︸}{x⊙\cdots ⊙x}}=\sum_{n}a_{n}\underset{\text{n-times}}{\underset{︸}{\hat{x}*\cdots *\hat{x}}}, \end{array} \end{array}$$

where *ϕ* is acting pointwise on the components of *x*, $Fx=\hat{x}$, and the RHS is a weighted sum of self-convolutions. Christian et al. (2021) show that repeated convolutions broaden the frequency spectrum by adding higher frequency components corresponding to large coefficients *a*_*n*_, an effect they call “blue shift”. A visual illustration of the blue-shift effect is shown in Figure 1 where we considered a one dimensional sinusoidal stimulus *s* filtered by softplus, tanh, ReLU, and hardtanh non-linearities. Additional to the blue shift effect (harmonic distortion), we also see the impact of intermodulation.

Let us now consider a more complex non-linear function such as a trained neural network. In this case, the non-linear distortion induced by the network will be manifested in its representation space and therefore in its decision making.

**Figure 2:**
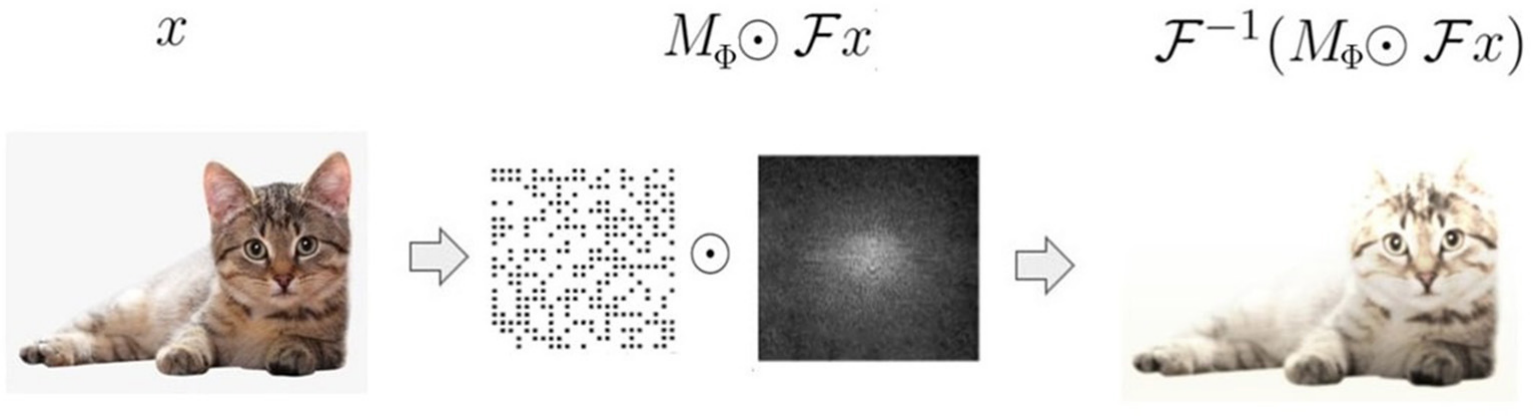
A schematic of the preprocessing layer defined by the mask: the input *x* is transformed into the Fourier domain where it is filtered with a learnable mask *M*_Φ_. $M_{\Phi }⊙Fx$ is then remapped into the pixel domain through the inverse Fourier transform $\bar{x}=F^{-1}\left(M_{\Phi }⊙Fx\right)$.

As mentioned above, one of the purposes of this work is to propose an algorithm to identify the *essential input frequencies in a trained ANN's decisions*. To this end, let us consider an image dataset $X={\left\{\left(x_{i},y_{i}\right)\right\}}_{i=1}^{N}$, where $x_{i}\in {\mathbb{R}}^{d\times d}$ denotes the *i*-th input image and *y*_*i*_∈ ℤ _*C*_ its associated label (*C* denotes the number of classes). We split X into a training set $X_{T}$ and a validation set $X_{V}$. We obtain the masks *via* the following optimization algorithm: we first pre-train a network Φ on $X_{T}$ with the objective of solving a classification task. We subsequently freeze the weights of Φ and attach a pre-processing layer whose weights are the entries *m*_*ij*_ of a mask matrix $M_{\Phi }\in {\mathbb{R}}^{d\times d}$. This layer acts as follows: for every $x\in X_{V}$ we modulate its Fourier transform Fx by computing the product $M_{\Phi }⊙Fx$, where ⊙ indicates the Hadamard product. We next compute the inverse Fourier transform $\bar{x}=F^{-1}\left(M_{\Phi }⊙Fx\right)$, which is then fed into the network (see Figure 2). Finally, we learn the mask *M*_Φ_ by solving the optimization problem


(2) $$\begin{array}{l} M_{\text{Φ}}(\lambda ,p)={\text{argmin}}_{M_{\text{Φ}}}\sum_{x\in X_{V}}e^{{\left[ℒ(\text{Φ}(\bar{x}),y)-ℒ(\text{Φ}(x),y)\right]}^{2}}+\lambda \|M_{\text{Φ}}{\|}_{p},\;\\ \text{ λ}\in {\mathbb{R}}_{+}, \end{array}$$

where Φ denotes the pre-trained network, λ||*M*_Φ_||_*p*_ is a regularization term penalizing the *p*-norm of the learned mask, and L is the loss function associated with the classification task. The first term in Equation (2) enforces an *invariance* in the loss with respect to the transformation $x\mapsto \bar{x}$ induced by the mask. The latter is key because we are expecting the desired frequencies to be revealed when there is no change in the loss L and maximal change in the *p*-norm of the mask *M*_Φ_. In other words, the mask is determined by a *symmetry operation in the Fourier space of the input with minimal* *p-norm*. A solution to Equation (2) is a mask *M*_Φ_ addressing the question: which frequencies are essential in this trained ANN's decision making? Such masks, obtained for various data augmentation choices reveal the frequencies associated with each particular choice.

At this point, we note that the mask is learned on the validation set $X_{V}$ and not on the training set $X_{T}$. This is because we are interested in exploring the minimal set of frequencies preserving the *generalization* power of Φ. Moreover, we tested the stability of our mask generation algorithm across different runs. This is crucial since it attests to the reliability of our qualitative and quantitative analyses. We also note that masks can be obtained for single images, simply considering a single $x\in X_{V}$ in Equation (2) instead of the full validation set or a subset of it (e.g., class-specific masks, see **Figure 5**).

### 2.2. Dataset and Simulations

Our data consisted of 6, 644 image/label pairs from 5 classes of ImageNet (Deng et al., 2009). Four thousand seven hundred and ten of those pairs belong to our training set $X_{T}$ and the remaining 1, 934 pairs belong to our validation set $X_{V}$. For simplicity, we choose grayscale versions of our dataset images, though our method can be applied for any number of input channels. Our images were centered with respect to the mean and standard deviation of $X_{T}$.

We initially trained VGG11 (Simonyan and Zisserman, 2015) and ResNet18 (He et al., 2016) baseline models on $X_{T}$ using the Pytorch framework. The performance of the models on the task was comparable and the results produced qualitatively similar. We therefore opted to present only the results obtained for VGG11. However, the interested reader can implement both models *via* the GitHub repository provided. For each subsequent training run we varied the type of data augmentation used for pre-processing (adversarial examples, random scales (for scaling factors in the interval [0.5, 1.5]), random translations (max absolute fraction for horizontal and vertical translations in [0.4, 0.4]), random rotations (for angles in [0, π])).

Each of the 5 networks in total was trained using the Adam optimizer (Kingma and Ba, 2015) and a maximum learning rate of 10^−3^. The learning rate of each learnable parameter group was scheduled according to the one-cycle learning rate policy with a minimum value of 0 (Smith, 2017). We found that this set of hyperparameter choices allowed us to achieve stable training for all our models. We trained each model for a maximum of 50 epochs and eventually evaluated our models on the validation set $X_{V}$. We finally saved the weight-state of each model that achieved the minimum Cross Entropy loss within the chosen interval of epochs. For each of our pre-trained networks, we learn its corresponding Fourier mask according to the algorithmic process presented in Section 2.1. We use ℓ_1_-regularization on the norm of the mask to enforce sparsity. We train masks on both the whole of $X_{V}$ but also for single images. Each scheme required its own hyperparameter tuning, which by simple grid search revealed the choices of λ = 0.2, 0.07 for masks on $X_{V}$ and masks for single images, respectively. In the next section, we present masks for every data augmentation scheme we chose as well as their respective differences. For a given set of masks, we center the mask differences around the origin. This helps with the interpretation of the masks without altering the geometry of the particular set.

## 3. Results

Adversarial training can be seen as a type of data augmentation where the inputs are augmented with adversarial examples (Goodfellow et al., 2014) to increase robustness to adversarial attacks. Here, we test the commonly accepted hypothesis that adversarially trained models need low frequency features for robustness. We do so by comparing the Fourier mask learned for a vanilla network Φ_*N*_ with that of an adversarially trained network Φ_*A*_ when the learning occurs over the whole validation set. Specifically, we compare a naturally trained VGG11 with an adversarially trained one using the *torchattacks* library (Kim, 2020) and a Projected Gradient Descent attack (PGD). Caro et al. (2020) has shown the frequency structures of adversarial attacks are similar across different adversarial attacks. Therefore, although the set of potential choices one can explore is vast, in this work we focus on PGD for simplicity. Besides the mask difference we also compute the radial and angular energy of each mask by considering radial and angular partitions of the frequency domain (**Figure 4**). We then test if the same low-frequency preference hypothesis holds true in the case of common data augmentations. To gain some intuition, let us consider a simple one-layer network whose representation is given by Φ(*x*) = σ〈*w, x*〉, where σ:ℝ → ℝ is a non-linear function, *x, w* ∈ ℝ ^*d*^, and ℓ:ℝ → ℝ_+_ is a cost function. We consider data augmentations generated by a group of transformations $G:=\left\{g_{\theta }:\theta \in \mathbb{R}\right\}\subset {\mathbb{R}}^{d\times d}$. The augmented loss can now be expressed as


$$\begin{array}{l} ℒ(w)=\frac{1}{N}\sum_{i=1}^{N}\int \ell \left(\sigma \langle w,g_{\theta }x_{i}\rangle ;y_{i}\right)d\theta \;\\ \;=\frac{1}{N}\sum_{i=1}^{N}\int \ell \left(\sigma \langle g_{\theta }^{*}w,x_{i}\rangle ;y_{i}\right)d\theta ,\;(x_{i},y_{i})\in X, \end{array}$$

where the second equality holds because $\langle w,g_{\theta }x_{i}\rangle =\langle g_{\theta }^{*}w,x_{i}\rangle$ and g^*^ denotes the adjoint. We note that in this context the loss function is *invariant to* *G* *transformations of the weights*, i.e., $L\left(g_{\theta }w\right)=L\left(w\right)$ for any *g*_θ_∈ *G* (the proof of this statement relies on simple properties of group transformations, see Chen et al., 2020). Here, we explore the impact such an invariance of the loss function has on the learned Fourier masks. The reasoning is as follows: updating the weights of an ANN is achieved through gradient descent, i.e., $\Delta w_{t}=-\alpha \nabla_{w}L\left(w_{t}\right)$, where *w*_*t*_ denotes the weights of the network at iteration *t* and α ∈ ℝ^+^ is the learning rate. The frequency content of the gradient of the loss at iteration *t* affects the frequency content of the weights. In turn, the latter determine the input frequencies the network is analyzing and thus will determine the mask. In other words, the frequency content of the loss, as well as how it is modified by different data augmentations, will impact the frequency content observed in the mask.

Let us consider a simple one dimensional example (*d* = 1) and the translation operator. In this case the loss L is *invariant to translations of the weights*, i.e.,


$$L\left(T_{t}\left(w\right)\right)=L\left(w\right),\;\forall t\in \mathbb{R},$$

where *T*_*t*_:ℝ → ℝ is the translation operator defined as *T*_*t*_(·) = ·−*t*. For $x_{i}\in X$ and *t* ∈ ℝ, let *q*_*i*_(·): = ℓ(σ(*T*_*t*_(·)*x*_*i*_);*y*_*i*_). Then the Fourier transform of L yields


$$\begin{array}{l} ℱ(ℒ)(\gamma )=\frac{1}{N}\sum_{i=1}^{N}\int_{-\infty }^{+\infty }ℱ(q_{i})(\gamma )e^{-2\pi i\gamma t}dt\;\\ \;=\frac{1}{N}\sum_{i=1}^{N}\delta (\gamma )ℱ(q_{i})(\gamma )=\frac{1}{N}\sum_{i=1}^{N}ℱ(q_{i})(0) \end{array}$$

where we used the translation property of the Fourier transform and δ denotes the Dirac delta. This simple example illustrates the effect of the translation operator on the loss L, i.e., a shift toward low frequencies (in this case a full shift of all frequencies to the DC component, the only non-zero component in the above equation). Note that an augmentation with all possible translations is not realistic. However, even a finite range of translations in the interval *t* ∈ [−*a, a*], for a sufficiently large *a*, will produce a similar effect. Indeed, we have


$$\begin{array}{l} ℱ(ℒ)(\gamma )=\frac{1}{N}\sum_{i=1}^{N}\int_{-\infty }^{\infty }ℱ(q_{i})(k)\chi_{\left[-a,a\right]}(t)e^{-2\pi i\gamma t}dt\;\\ \;=\frac{2a}{N}\sum_{i=1}^{N}\text{sinc}(2\pi \gamma a)ℱ(q_{i})(\gamma ) \end{array}$$

where χ denotes the characteristic function. Thus, the impact of averaging over an interval of translations on L is to dampen its frequencies with a sinc function profile, i.e., a frequency re-weighting with a *bias for low frequencies*. However, we stress that the above argument is developed with a 1-layer network in mind. The effect of data-augmentation with respect to random translations viewed through a deep network is expected to be more intricate.

### 3.1. Masks Generated for the Whole Dataset

**Figure 3:**
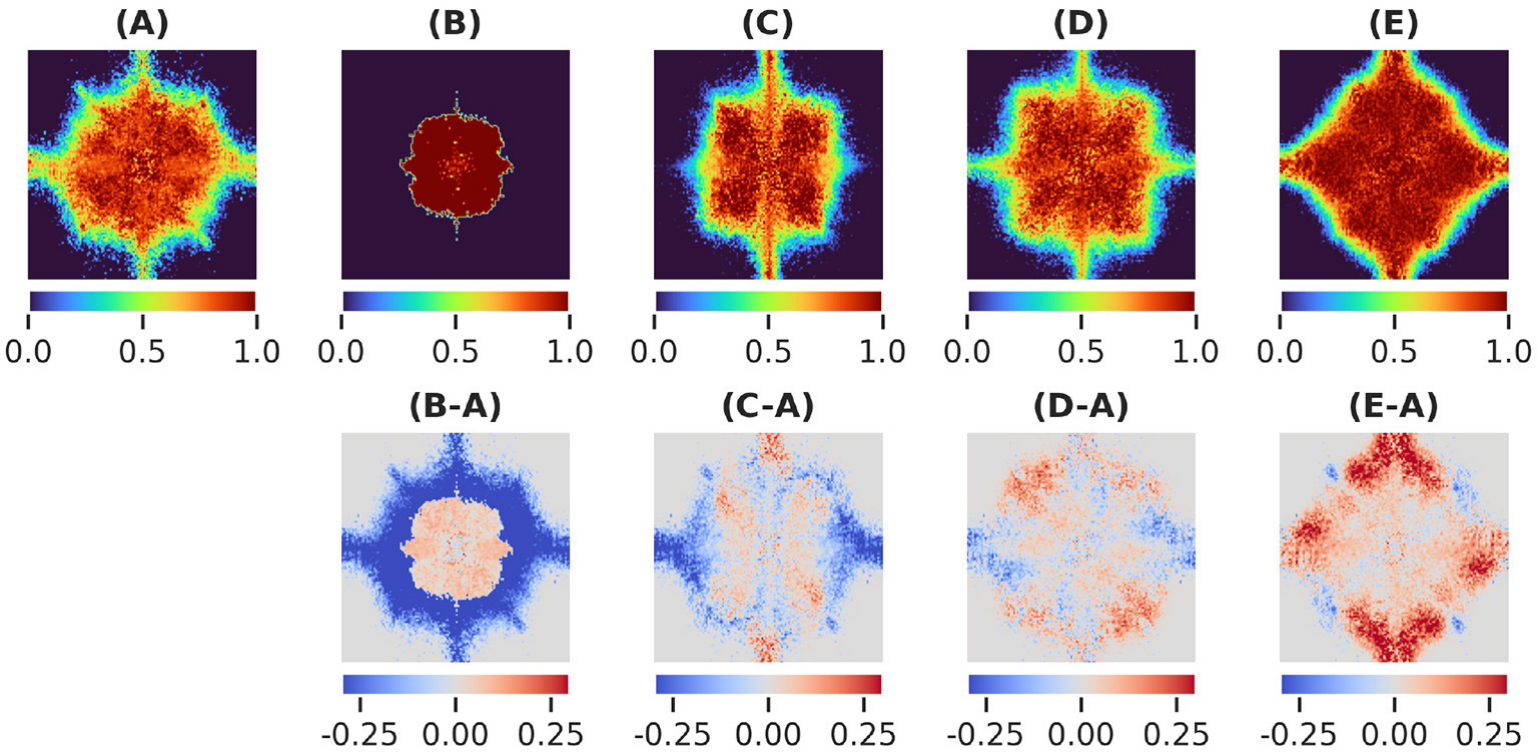
**(A)** Learned masks for the vanilla network *M*_*N*_, **(B)** Adversarially trained network *M*_*A*_, **(C)** scale-invariant network *M*_*S*_, **(D)** translation-invariant network *M*_*T*_ , **(E)** rotation-invariant network *M*_*R*_ and their differences.

We generated masks over $X_{V}$ for networks trained to be robust to adversarial examples, random scales, translations, and rotations. The masks in Figure 3 and their differences reveal how distinct frequency biases depend on the type of data augmentation. We also note how model performance is minimally altered by the introduction of the mask layer Table 1.

**Table 1:** Model performance (%) with and without the mask layer.

	***M***_***N***_	***M***_***A***_	***M***_***S***_	***M***_***T***_	***M***_***R***_
Standard	89.56	79.62	86.62	85.86	68.47
Masked	89.20	78.97	86.72	85.35	68.17

*All accuracies are reported on the non-augmented validation set*.

**Figure 4:**
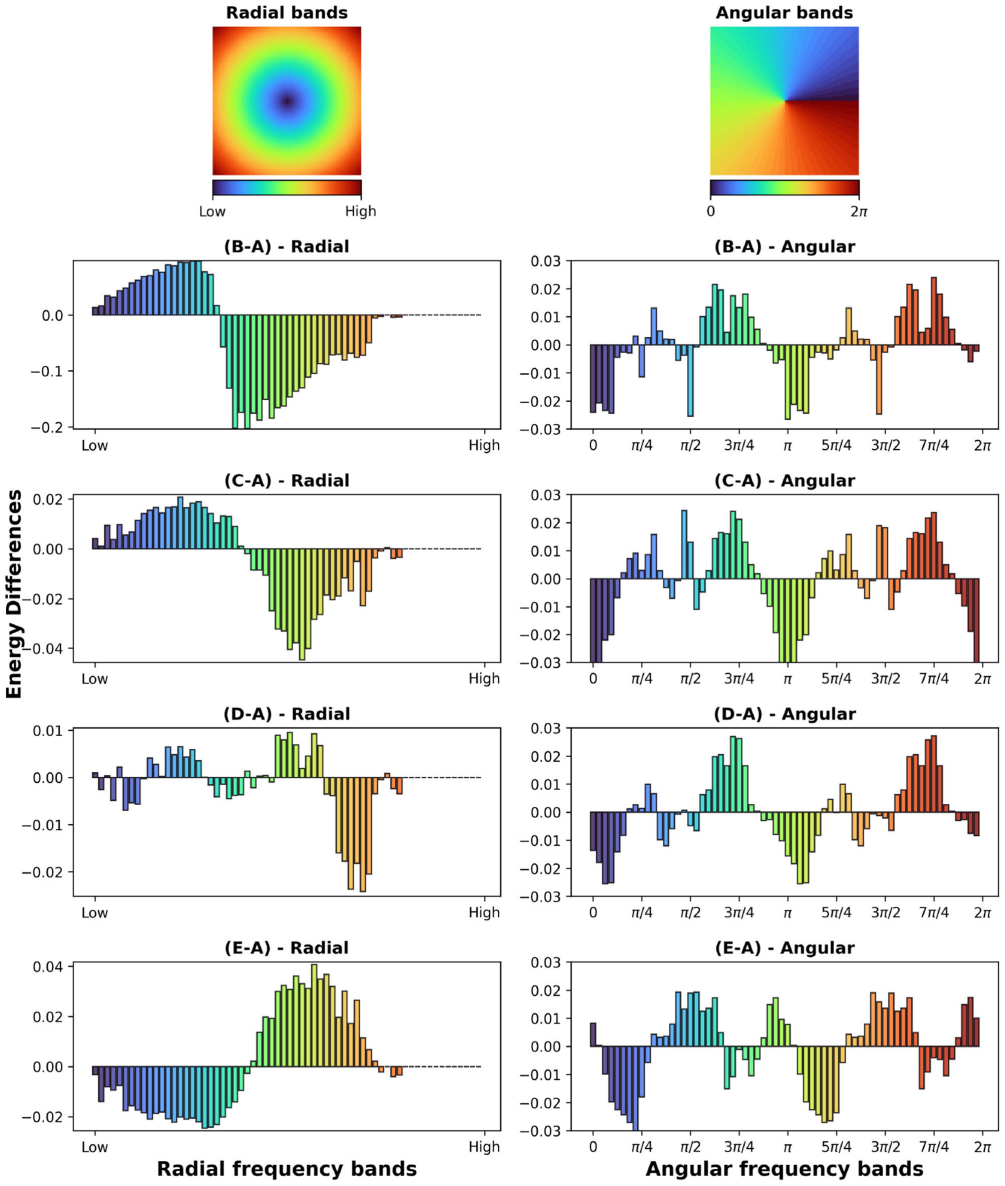
(First row) Radial and angular partitions of the Fourier domain; Energy differences **(B-A, C-A, D-A, E-A)** in radial and angular directions for the augmentations in Figures 3(B–E).

In the case of adversarial augmentation there exists a net bias toward low frequencies as shown by the difference between the masks generated by the vanilla and adversarial trained network in Figure 3B-A. This is further confirmed by the radial energy difference in Figure 4(B-A)-radial, while the angular energy difference in Figure 4(B-A)-angular shows that the redistribution of the frequencies occurs anisotropically.

In the case of common augmentations our results exhibit contrasting effects in the Fourier masks. While the redistribution of the mask frequencies seems to be directionally-dependent (Figure 4B(C-A),(D-A),(E-A)-angular), only robustness to scales endows the net with a bias toward low frequencies (Figure 4(C-A)-radial). For translations the mask implies a less clear effect (Figure 4(D-A)-radial), where a mixed behavior is present for mid and low frequencies. Interestingly, in the case of rotational robustness, Figure 4(E-A)-radial shows a high frequency bias.

### 3.2. Masks Generated for Single Images

**Figure 5:**
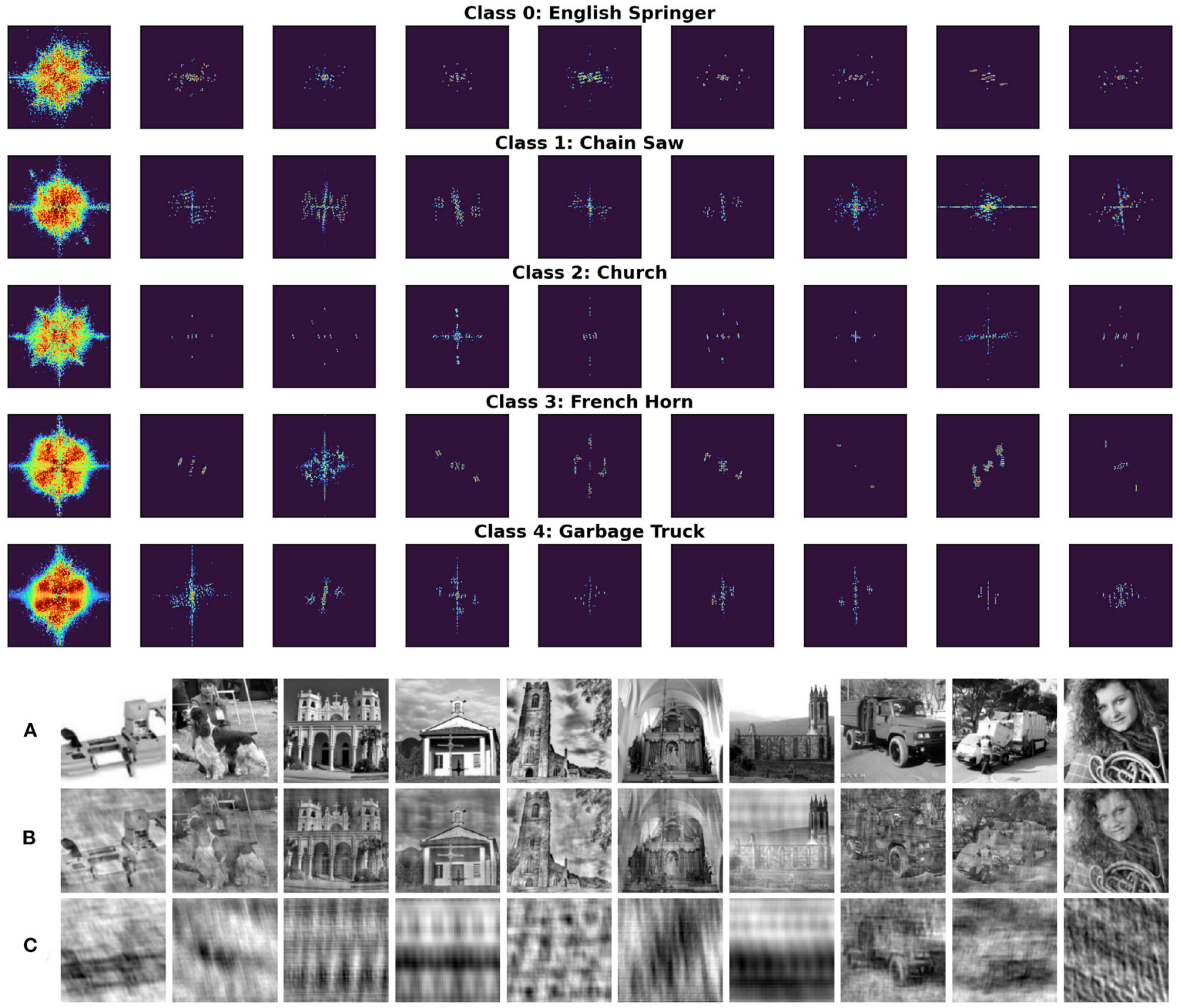
**(Top)** Randomly sampled single image masks divided by class. The first column corresponds to masks trained on all the images belonging to each class separately. The colormap is the same as that of Figure 3. **(Bottom) (A)** Randomly sampled images; **(B)** filtered by their complementary masks $M_{N,x}^{c}$; **(C)** the same images filtered by their associated Fourier masks *M*_*N,x*_.

To further investigate the nature of adversarial robustness and how it is related to a network's generalization properties in the frequency domain we generated Fourier masks *M*_*N,x*_ for each *correctly-classified* image *x* in the validation set *X*_*V*_. Moreover, for each such image *x* we consider its adversarial counterpart so that all adversarial examples are miss-classified. Figure 5 (top) shows such masks randomly sampled for images in all 5 data classes trained with respect to the vanilla network Φ_*N*_.

It is worth noting that the masks are very sparse, i.e., very few frequencies are essential for preserving the prediction of the pretrained network. Additionally, for every mask *M*_*N,x*_, we also consider its complementary mask $M_{N,x}^{c}$ defined as


$$\begin{array}{l} M_{N,x}^{c}(i,j)=\left\{ \begin{array}{l} 1,\;M_{N,x}(i,j)<10^{-8}\;\\ 0,\text{ otherwise.} \end{array}\right. \end{array}$$

**Figure 6:**
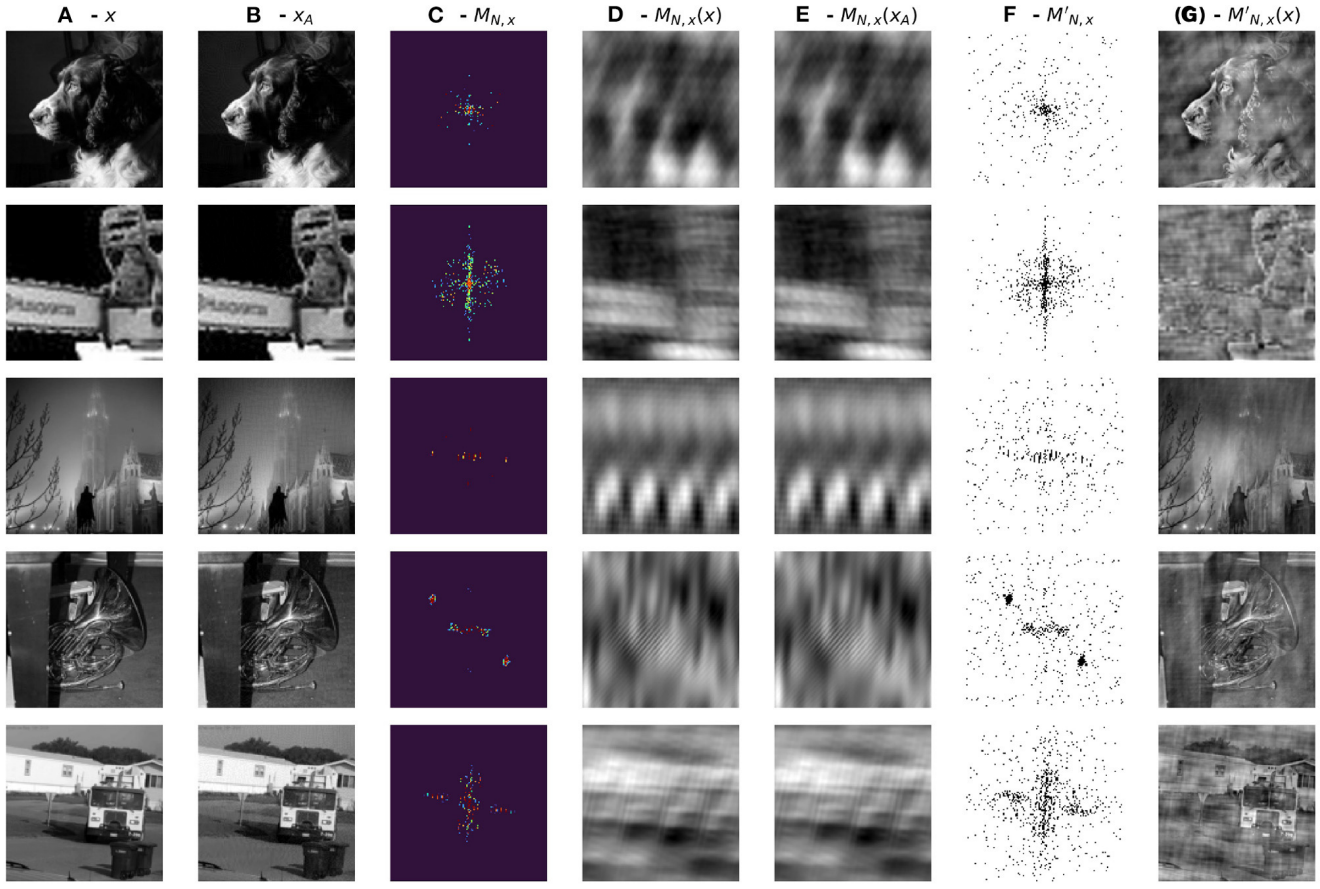
**(A)** Correctly classified image $x\in X_{V}$; **(B)** Adversarial Image *x*_*A*_; **(C)** *M*_*N,x*_ - learned mask from vanilla network; **(D)** *M*_*N,x*_ - filtered image *x*; **(E)** *M*_*N,x*_ - filtered image *x*_*A*_; **(F)** $M_{N,x}^{c}$ - binary complement of *M*_*N,x*_; **(G)** $M_{N,x}^{c}$ - filtered image *x*.

Filtering an image with its complementary mask $M_{N,x}^{c}$ does not compromise our ability to recognize the filtered image (Figure 5B, Bottom). On the contrary, filtering with the mask *M*_*N,x*_ renders the image unrecognizable (Figure 5C, Bottom). Filtered images resemble texture-like patterns. Interestingly, recent work by Geirhos et al. (2019) shows how ImageNet-trained CNNs are strongly biased toward recognizing textures rather than shapes. Figure 6 further confirms these results extending them to the case of adversarial images showing the masks learned from the vanilla and adversarially trained networks and their corresponding filtered images. Surprisingly, performance drops drastically (~ 45% decrease) for images filtered by complementary masks $M_{N,x}^{c}$. Additionally, filtering adversarial examples using masks generated from original images reverses the effect of the attack in approximately 60% of validation samples. We unpack this information in Table 2 below.

**Table 2:** *M*_*N,x*_(*X*) and *M*_*N,x*_(*X*_*A*_) denote original images/adversarial images filtered by masks trained for original images from the vanilla network.

	**Data x**	***Adv. Data x*_*A*_**	***M***_***N,x***_ **(*x*)**	***M*** **_*N,x*_(*x*_*A*_)**	** $M_{N,x}^{c}\left(X\right)$ **
Model accuracy	100%	0%	100%	58.83%	54.4%

*$M_{N,x}^{c}\left(X\right)$ denotes the set of original images filtered by the complementary masks of M_N,x_*.

We think this is an interesting result since

- The increase in performance when testing on *M*_*N,x*_(*x*_*A*_) provides strong evidence that the attack mostly relies on frequencies not present in the mask *M*_*N,x*_.
- The drop in performance when testing on $M_{N,x}^{c}\left(x\right)$ implies that the frequencies learned by each individual mask are not only sufficient but also necessary for the task.

**Figure 7:**
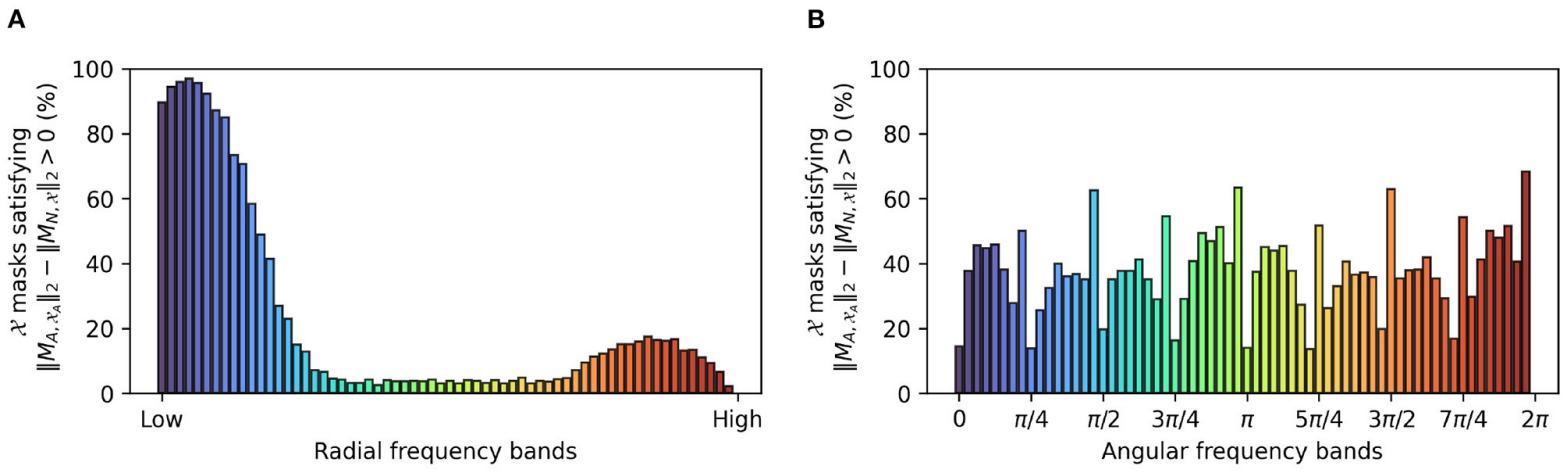
Comparison of X-trained masks ($M_{N,X}$) obtained for the vanilla network and $X_{A}$-trained masks ($M_{A,X_{A}}$) obtained for an adversarially trained network: the bars illustrate the percentage of masks for which the per-band energy [radial **(A)** and angular **(B)**] in in $M_{A,X_{A}}$ exceeds that of $M_{N,X}$.

Further confirming a low frequency bias in adversarially trained networks, Figure 7 shows the percentage of perturbed images for which the per-band energy (radial or angular) of their corresponding masks $M_{A,X_{A}}$ exceeds that of the masks $M_{N,X}$ generated from the non-perturbed examples. Figure 7 confirms that lower frequencies are preferred for a robust representation.

Finally, upon visual inspection of the learned single-image masks we also suspected that such masks exhibit class-specificity. We tested this hypothesis by learning a linear classifier on the a uniformly balanced set of single image masks $\left\{M_{N,x}|x\in X_{V}\right\}$. We considered 85% of the masks to be our training set for this task and later tested the linear classifier on the remaining 15% of single-image masks. Table 3 below confirms that the essential frequencies for this network's generalization performance are class-specific. We also tested the robustness of this experiment by randomly shuffling the labels of the learned masks and testing if a linear classifier is still able to separate the masks based on their new label assignment. Table 3 shows this is not the case and the results suggest that linear separability of the masks is due to their geometry and not the representation power of the linear classifier.

**Table 3:** Training a linear classifier to separate single-image masks trained on the test images of *X*.

	***M*_*N,X*_**	***M*_*N,X*_**
	**True labels**	**Shuffled labels**
	**(%)**	**(%)**
Training accuracy	93.22	19.50
Test accuracy	83.87	16.42

**Figure 8:**
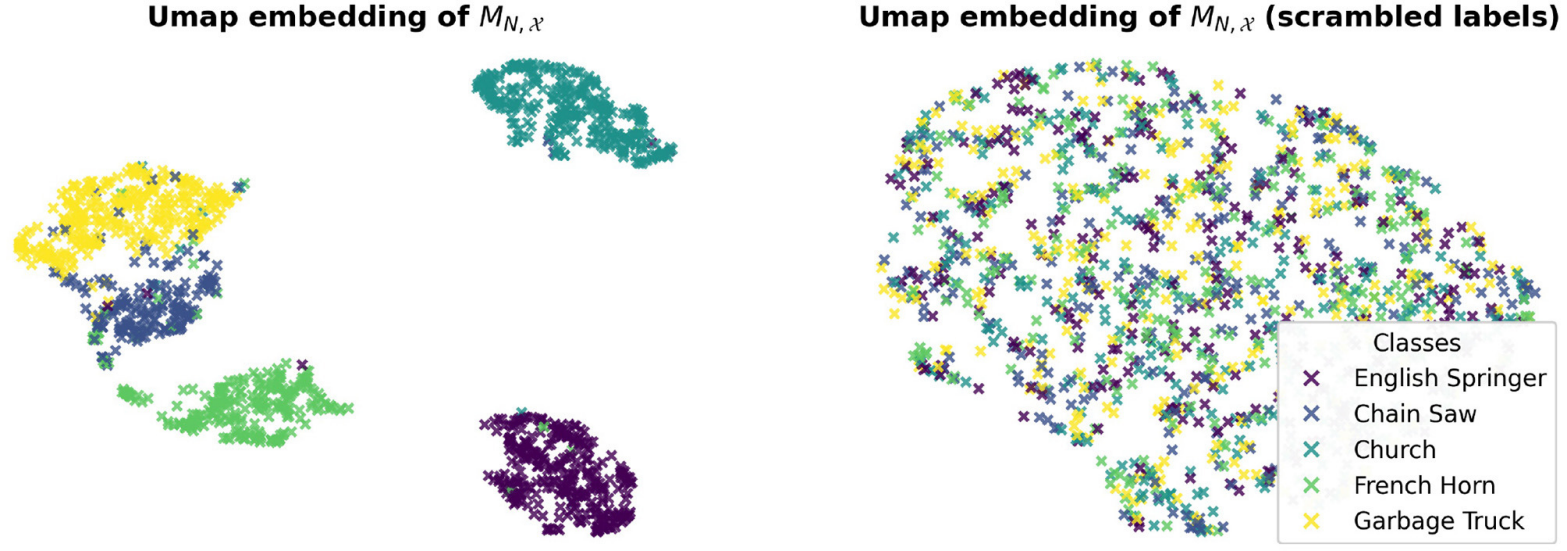
Clustering of the output of a linear classifier learned to separate single image Fourier masks. $M_{N,X}$ indicates the set of single image masks computed for the correctly classified test images in X.

We visually illustrate these results by performing a manifold analysis of the learned masks using UMAP (McInnes et al., 2018) for dimension reduction and visualization. Interestingly, we found that the masks are linearly separable and that the linear network responses cluster (Figure 8).

## 4. Discussion and Conclusions

In this work, we proposed a simple yet powerful approach to visualize the essential frequencies a trained network is using to solve a task. Our strategy consists of learning a frequency modulatory mask characterized by two critical properties:

- It defines a symmetry in the Cross Entropy loss, i.e., it does not alter the pretrained model's predictions.
- It has minimal ℓ_*p*_-norm, which for *p* = 1 guarantees the preservation of performance while promoting sparsity in the mask.

Using our method we tested the common hypothesis that adversarially trained networks prefer low frequency features to achieve robustness. We also tested if this hypothesis holds true for common data augmentations such as translations, scales, and rotations.

In the case of adversarial augmentation, our results confirm the low frequency bias hypothesis. However, they also highlight that the frequency redistribution due to the augmentation is highly anisotropic. In the case of common data augmentations instead, our results show how the frequency reorganization depends on the type of augmentation.

In the case of adversarial training we also run a single image analysis to detect the frequencies useful for adversarial robustness and those responsible for adversarial weakness. Here too, masks learned on adversarially trained networks concentrate more toward lower frequencies compared to those learned on vanilla networks. Furthermore, the analysis showed that only a sparse, class-specific set of frequencies is needed to classify an image. Surprisingly, mask-filtered images in this case are not recognizable and resemble texture-like patterns, supporting the idea that ANNs use fundamentally different classification strategies from humans to achieve robust generalization (Geirhos et al., 2019).

To our knowledge the use of a learned mask to characterize a network's crucial property such as robust generalization has not been proposed before. The interpretation of the masks provides us with a detailed geometrical description of directional and radial biases in the frequency domain as well as with quantifiable differences between various training schemes.

Our analysis can be extended to other architectural or optimization specifics, e.g., explicit regularizations, different optimizers/initializations, etc. The same mask approach can be employed to modulate the phase and modulus in the Fourier transform of the data. Our method effectively opens up many directions in the investigation of a network's implicit frequency bias. Future research directions will also include a natural generalization of our approach where the image features are learned, rather then fixed to be of the Fourier type.

## Data Availability Statement

Publicly available datasets were analyzed in this study. This data can be found here: https://github.com/fastai/imagenette. Code is available at https://github.com/nkarantzas/FourierMasks.

## Author Contributions

NK and FA conceived the conceptualized framework and wrote the first draft. NK and EB trained and analyzed models. AP, JO, AT, and XP provided the feedback along the way. AT, AP, and XP provided the funding. All authors revised, edited and provided comments on the final manuscript and contributed to the article and approved the submitted version.

## Funding

This research was supported by the Intelligence Advanced Research Projects Activity (IARPA) via Department of Interior/Interior Business Center (DoI/IBC) contract no. D16PC00003. The US Government is authorized to reproduce and distribute reprints for governmental purposes notwithstanding any copyright annotation thereon. This work is also supported by the Lifelong Learning Machines (L2M) Program of the Defense Advanced Research Projects Agency (DARPA) via contract number HR0011-18-2-0025 and R01 EY026927 to AT and by NSF NeuroNex grant 1707400.

## Author Disclaimer

The views and conclusions contained herein are those of the authors and should not be interpreted as necessarily representing the official policies or endorsements, either expressed or implied, of IARPA, DoI/IBC or the US Government.

## Conflict of Interest

The authors declare that the research was conducted in the absence of any commercial or financial relationships that could be construed as a potential conflict of interest.

## Publisher's Note

All claims expressed in this article are solely those of the authors and do not necessarily represent those of their affiliated organizations, or those of the publisher, the editors and the reviewers. Any product that may be evaluated in this article, or claim that may be made by its manufacturer, is not guaranteed or endorsed by the publisher.
